# MicroRNA-365 regulates IL-1β-induced catabolic factor expression by targeting HIF-2α in primary chondrocytes

**DOI:** 10.1038/s41598-017-18059-6

**Published:** 2017-12-20

**Authors:** Hyun Sook Hwang, Su Jin Park, Mi Hyun Lee, Hyun Ah Kim

**Affiliations:** 1 Division of rheumatology, Department of Internal Medicine, Hallym University Sacred Heart Hospital, Kyunggi, 431-070 Korea; 2 Institute for Skeletal Aging, Hallym University, Chunchon, 200-702 Korea

## Abstract

Endothelial Per-Arnt-Sim domain protein-1/hypoxia-inducible factor-2α (EPAS-1/ HIF-2α) is a catabolic transcription factor that regulates osteoarthritis (OA)-related cartilage destruction. Here, we examined whether microRNA-365 (miR-365) affects interleukin (IL)-1β-induced expression of catabolic factors in chondrocytes via regulation of HIF-2α. MiR-365 levels were significantly decreased in human OA cartilage relative to normal cartilage. Overexpression of miR-365 significantly suppressed IL-1β-induced expression of HIF-2α in human articular chondrocytes. Pharmacological inhibition of various IL-1β-associated signaling pathways revealed mitogen-activated protein kinase and nuclear factor-κB as the primary pathways driving IL-1β-mediated decreases in miR-365 and subsequent increase in HIF-2α expression. Using a luciferase reporter assay encoding the 3′ untranslated region (UTR) of human HIF-2α mRNA, we showed that overexpression of miR-365 significantly suppressed IL-1β-induced up-regulation of HIF-2α. AGO2 RNA-immunoprecipitation (IP) assay demonstrated that miR-365 and HIF-2α mRNA were enriched in the AGO2-IP fraction in miR-365-transfected primary chondrocytes compared to miR-con-transfected cells, indicating that HIF-2α is a target of miR-365. Furthermore, miR-365 overexpression significantly suppressed IL-1β-induced expression of catabolic factors, including cyclooxygenase-2 and matrix metalloproteinase-1, -3 and -13, in chondrocytes. In pellet culture of primary chondrocytes miR-365 prevented IL-1β-stimulated extracellular matrix loss and matrix metalloproteinase-13 expression. MiR-365 regulates IL-1β-stimulated catabolic effects in human chondrocytes by modulating HIF-2α expression.

## Introduction

Osteoarthritis (OA) is a degenerative joint disease resulting from a variety of biomechanical and biochemical factors, leading to destruction of articular cartilage, synovial inflammation, and joint pain. Although OA is generally regarded as a non-inflammatory disease, various pro-inflammatory cytokines, such as interleukin (IL)-1β and tumor necrosis factor (TNF)-α, derived from the synovium and chondrocytes, have been shown to affect both the synthesis and destruction of cartilage matrix^1^.

IL-1β is synthesized as a precursor (pro-IL-1β) and activated through a proteolytic cleavage by caspase-1. Activated IL-1β binds to type I receptor (IL-1R1) and activates several signal transduction pathways, including protein kinase C, mitogen activated protein kinase (MAPK), NF-kB and activator protein 1 (AP-1)^2^. In addition, IL-1β induces matrix metalloproteinase (MMP) expression via inducible nitric oxide synthase (iNOS) induction and NO production in rabbit articular chondrocytes^3^. IL-1s were detected in the cartilage and synovial fluids of patients with rheumatoid arthritis (RA) and OA, suggesting that IL-1s are important mediators in the process of cartilage damage^4,5^. Under the condition of hypoxic culture, IL-1β-stimulated activation of MAPK, NF-kB, and AP-1 was significantly enhanced in articular chondrocytes compared with normoxia^6^. Recent studies have showed that IL-1β modulated two α subunits of hypoxia-inducible factor (HIF), HIF-1α and HIF-2α, in chondrocytes, suggesting a significance of HIF as a transcription regulator in chondrocytes during OA^7–9^.

HIF, a transcription factor composed of an α and β subunit, modulates oxygen-regulated gene expression by binding to hypoxia response elements at target gene loci. Activity is mediated through the dimerization of either HIF-1α or HIF-2α with the HIF-1β subunit, resulting in distinct transcriptional responses^9–11^. In particular, HIF-2α, also known as endothelial Per-Arnt-Sim domain protein-1 (EPAS-1), is an important regulator of cartilage catabolism under hypoxic conditions. HIF-2α is highly expressed in osteoarthritic cartilage and causes cartilage degradation by inducing expression of MMPs, a disintegrin and metalloproteinase with thrombospondin motifs (ADAMTS) 4, and nitric oxide synthase (NOS)-2^9^. Depletion of HIF-2α using HIF-2α-haplosufficient mice showed a reduction in cartilage degradation and osteophyte formation, suggesting that HIF-2α regulates the pathogenesis of OA^12^.

MicroRNAs (miRNAs), endogenous small non-coding RNAs ~22 nucleotides in length, are involved in the regulation of target genes via translational inhibition and/or target mRNA degradation^13^. Altered expression of various miRNAs has been observed in OA compared to normal cartilage, suggesting that miRNA expression may affect cartilage homeostasis^14^. Previously, we were able to demonstrate that miR-127-5p and miR-558, which are regulated by IL-1β in chondrocytes and downregulated in OA cartilage, are able to regulate MMP-13 and cyclooxygenase-2 (COX-2) expression, respectively^15,16^. Given these and other studies indicating a variety of miRNAs regulated by IL-1β in chondrocytes^17–19^, we applied a series of bioinformatics screening tools, including miRanda (http://www.microrna.org), TargetScan (http://www.targetscan.org), and PicTar (http://pictar.mdc-berlin.de/), to identify miRNAs that may target HIF-2α. From this effort we identified has-microRNA-365 (miR-365), a miRNA previously implicated in ADAMTS-1 expression in breast cancer cell-line, as a potential regulator of HIF-2α. Additionally, miR-365 has been shown to affect two cartilage matrix regulation genes, including *Runx1tl* (runt-related transcription factor 1; translocation to, 1) expression in brown fat differentiation, and *ACVR1/Alk-2*, which encodes a bone morphogenetic protein (BMP) type I receptor^20–27^. Recently, miR-365 was shown to be involved in chondrocyte differentiation through direct targeting histone deacetylase-4 (HDAC4)^28^. In a recent report, miR-365 was up-regulated in OA chondrocytes compared to normal cells, while its level was suppressed in response to hydrostatic pressure^29^.

In this study, we examined whether miR-365 inhibits IL-1β-stimulated catabolic responses via modulation of HIF-2α expression in human chondrocytes. Our results prove that HIF-2α is a target of miR-365, suggesting that miR-365 plays a crucial role in maintaining cartilage homeostasis.

## Materials and Methods

### Materials

Recombinant human IL-1β and antibodies to inducible nitric oxide synthase (iNOS), beta-actin (β-actin), MMP-1, and MMP-3 were purchased from R&D Systems (Minneapolis, MN, USA). Antibodies against COX-2, SAPK/JNK, phospho-SAPK/JNK (Thr183/Tyr185), p38 MAPK (p38), phospho-p38 (Thr180/Tyr182), p44/42 MAPK (Erk1/2), phospho-ERK1/2 (Thr202/Tyr204), nuclear factor of kappa light polypeptide gene enhancer in B-cells inhibitor alpha (IκBα), phosphor-IκBα (Ser32), NF-κB p65, phospho-NF-κB p65 (Ser536) and lamin B1 were purchased from Cell Signaling Technology (Danvers, MA, USA). Horseradish peroxidase-conjugated secondary antibodies and anti-HIF-2α antibody were obtained from Santa Cruz Biotechnology (Santa Cruz, CA, USA) and Abcam (Cambridge, UK), respectively. MiR-365 and control microRNA (miR-con) were purchased from Bioneer (Daejeon, Korea). Primers, including HIF-2α 3′UTR and mutant HIF-2α 3′UTR, were obtained from Cosmogenetech (Seoul, Korea). SP600125, PD98059, SB203580, and Bay 11-7082 were obtained from Sigma-Aldrich (St. Louis, MO, USA).

### Collection of OA cartilages and chondrocytes

OA cartilage samples were obtained from the knee joints of OA patients [n = 10, 71.9 ± 7.1 years] at the time of total knee replacement surgery. Patient diagnoses were determined using the criteria set forth by the American College of Rheumatology. Normal cartilage samples were obtained from the femoral head of patients [n = 10, 70.1 ± 12.5 years] with femoral neck fractures and no known history of OA or RA. Sample was obtained only from grossly normal-looking cartilage. The collection and use of human tissue samples was reviewed and approved by the Institutional Review Board of Hallym University Sacred Heart Hospital, Anyang, Korea (approval number 2013-I022). All patients provided written informed consent. All methods were performed in accordance with the relevant guidelines and regulations of Hallym University and were approved by its ethical committee.

Chondrocytes were isolated by carefully dissecting articular cartilage from a relatively lesion-free area, followed by sequential digestion with a protease (8 μg/mL) from *Streptomyces griseus* for 1 h and with collagenase (4 μg/mL) from *Clostridium histolyticum* and hyaluronidase (0.2 μg/mL) from bovine testes (Sigma-Aldrich) for 2 h. Chondrocytes were maintained in monolayer culture in Dulbecco’s modified Eagle’s medium (DMEM) containing 10% fetal bovine serum (FBS) and 1% penicillin/streptomycin at 37 °C in a humidified atmosphere of 5% CO_2_ and 95% air. First-passage cultured chondrocytes were used within 7–10 days after seeding.

SW1353 cells, human chondrogenic cell line, were cultured in DMEM containing 10% FBS and 1% penicillin/streptomycin at 37 °C in a humidified atmosphere of 5% CO_2_ and 95% air for pellet culture.

To examine the effect of MAPK and NF-κB signaling pathway on miR-365 and HIF-2α expression, chondrocytes were pretreated with SP600125 (10 µM), PD98059 (10 µM), SB203580 (1 µM), and Bay 11-7082 (5 µM) 2 h prior to stimulation with IL-1β (1 ng/mL) for 6 h.

### Quantitative real-time reverse transcription-polymerase chain reaction (qRT-PCR) to detect the expression of HIF-2α and miR-365

For isolation of total RNA from cartilage tissues, minced cartilage samples were ground to a fine powder in liquid nitrogen, and total RNA was isolated from chondrocytes using TRIzol reagent. For quantification of HIF-2α gene expression, cDNA was synthesized from 2 µg of RNA using Moloney murine leukemia virus reverse transcriptase (Promega, Madison, WI, USA). The qRT-PCR reactions contained SYBR Green PCR master mix, forward and reverse primer for HIF-2α, and an equal amount of cDNA from each sample. PCR amplification was performed under the condition of denaturation (95 °C for 15 sec) and anneal/extension (60 °C for 1 min) step for 40 cycles using a StepOnePlus real-time PCR system (Applied Biosystems). Glyceraldehyde 3-phosphate dehydrogenase (GAPDH) was used as an internal control. Primer sequences were as follows: HIF-2α forward 5′-GAG-GGT-TTC-ATT-GCT-GTG-GT-3′, HIF-2α reverse 5′-CTC-ACG-GAT-CTC-CTC-ATG-GT-3′; GAPDH forward 5′-TGA-TGA-CAT-CAA-GAA-GGT-GGT-GAA-G-3′, reverse 5′-TCC-TTG-GAG-GCC-ATG-TGG-GCC-AT-3′.

Expression of mature miR-365 was quantified using a Taqman miRNA reverse transcription kit and miRNA-specific primers obtained from Applied Biosystems (Foster, CA, USA) according to the manufacturer’s instructions. Purified RNA was reverse transcribed using the Taqman miRNA reverse transcription kit (Applied Biosystems) and miRNA-specific stem-loop RT primers (Applied Biosystems). PCR mixtures for miRNA detection contained 2 μl RT products, 5 μl TaqMan Universal PCR master mix, 0.2 μM Taqman probe, and 10 μM primers. qRT-PCR was performed using a StepOnePlus real-time PCR system (Applied Biosystems). RNU6B was used as an internal control for miRNA detection.

### Transfection of miRNAs and small interfering RNAs (siRNA)

HIF-2α siRNA (si-HIF-2α; sense 5′-CGU-GAG-AAC-CUG-AGU-CUC-A-3′; antisense 5′-UGA-GAC-UCA-GGU-UCU-CAC-G-3′), control siRNA (si-con; sense 5′-CCU-ACG-CCA-CCA-AUU-UCG-U-3′; antisense 5′-ACG-AAA-UUG-GUG-GCG-UAG-G-3′) as a negative control, the mature form of has-miR-365 (miR-365; 5′-UAA-UGC-CCC-UAA-AAA-UCC-UUA-U-3′), and nonspecific microRNA (miR-con; 5′-CCU-ACG-CCA-CCA-AUU-UCG-3′) as a off-target control were purchased from Bioneer (Daejeon, Korea). The antisense inhibitor of miR-365 (anti-miR-365) was obtained from Applied Biosystems. Human chondrocytes were transfected with miR-con, miR-365, or anti-miR-365 at a concentration of 50 nM using Lipofectamine according to the manufacturer’s instructions. In brief, miRNA or siRNA were gently mixed with plus reagent diluted with Opti-MEM media. After the diluted miRNA or siRNA was added to Lipofectamine reagent diluted with Opti-MEM, the solution containing miRNA or siRNA was gently mixed and incubated for 15 min at room temperature to allow miRNA- or siRNA-liposome complexes to form. The medium containing miRNA- or siRNA-liposome complexes was drop-wise added onto the chondrocytes and the cells were incubated for 3 h at 37 °C. The cells were maintained with fresh medium for 48 h. The cells were then stimulated with IL-1β (1 ng/mL) for 6 or 24 h and used for qRT-PCR and Western blot assays, respectively. In addition, to examine the effect of HIF-2α knockdown on IL-1β-stimulated catabolic factor expression in miR-365-stimulated chondrocytes, si-HIF-2α and miR-365 were co-transfected into the chondrocytes. Briefly, si-HIF-2α and/or miR-365 were prepared by gently pipetting in Lipofectamine reagent diluted with Opti-MEM media. Co-transfection complexes were drop-wise added to the chondrocytes with gently rocking the culture plate. After 3 h incubation the culture medium was replaced by fresh medium. Co-transfected chondrocytes were stimulated with IL-1β (1 ng/mL) for 6 or 24 h as described above.

### Immunohistochemical analysis

Cartilage tissues were fixed in 4% paraformaldehyde, embedded in paraffin, and sectioned using a microtome. Sections were blocked with 3% bovine serum albumin at room temperature for 1 h and incubated with anti-HIF-2α antibody (1: 100 dilution) for 16 h at 4 °C. Sections were then rinsed, incubated with biotinylated secondary antibody (1:200 dilution) for 30 min, and treated with Vectastatin ABC reagent (Vector Laboratories, Burlingame, CA, USA) for 30 min, and visualized with 3,3′-diaminobenzidine for 8 min. To quantify the protein level of HIF-2α in normal and OA cartilages, total number of chondrocytes and the number of chondrocytes staining positive for HIF-2α were evaluated and results were expressed as the percentage of chondrocytes staining positive in 2 different fields per section.

### Enzyme-linked immunosorbent assay (ELISA)

Cell culture medium was collected to measure the level of secreted MMP-13. MMP-13 protein levels were quantified using a pro-MMP-13 immunosorbent assay according to the manufacturer’s instructions (R&D Systems). Briefly, 100 µL of standard solution and sample was added to each well of 96-well plate, which was coated with MMP-13 antibody. Biotin-labeled detection antibody (100 μL) was added to the wells for 1 h at 37 °C. After washing with wash buffer, avidin-horse radish peroxidase (HRP) conjugate was added to each well at room temperature for 30 min and substrate reagent was added to each well for 15 min. The reactions were finished by adding stop solution to each well. Optical density was measured at 450 nm using a Thermo Scientific Multiskan GO Microplate Spectrophotometer (Thermo Fisher Scientific, Waltham, MA, USA). The concentrations (pg/mL) of MMP-13 of the samples were calculated from the standard curve.

### Western blot analysis

Cell lysates were prepared with radioimmunoprecipitation assay (RIPA) lysis buffer (Biosesang, Kyunggi, Korea) and protein concentrations were quantified using bicinchoninic acid protein assay (Thermo Scientific, Rockford, IL, USA). Equal amount of proteins were resolved by 10% sodium dodecyl sulfate-polyacrylamide gel electrophoresis (SDS-PAGE) and electrotransferred to a polyvinylidene difluoride membrane (Bio-Rad Laboratories, Hercules, CA, USA). The membrane was blocked with 5% (w/v) nonfat milk in TBST (Tris buffered saline and 0.1% Tween 20) and incubated with primary (1: 1,000 dilution) and secondary antibodies (1: 5,000 dilution). The membrane was developed using an enhanced chemiluminescence kit (Santa Cruz Biotechnology).

### Luciferase construct and reporter assay

The 3′UTR sequence (5′-UGUUGCCCUGGCAUUAAGGGCAUUUUACCCUUGCAUUU-3′) of HIF-2α containing the putative miR-365 binding site were obtained from gene sequence databases, and used to design the wild-type HIF-2α 3′UTR-Luc plasmid (HIF-2α 3′UTR). Briefly, HIF-2α 3′UTR was amplified by PCR using forward primer 5′-CTC-GAG-AAA-GCA-CAT-TGG-GCC-AAC-TAT-TTA-GTA-AGC-CC-3′ and reverse primer 5′-GCG-GCC-GCT-GAA-GCT-TGG-AAT-ATT-TTT-CAG-TG-3′, and cloned into a multiple cloning region in a psi-CHECK-2 vector (Promega). For construction of the mutant HIF-2α 3′UTR-Luc plasmid (mut HIF-2α 3′UTR), a mutant HIF-2α 3′UTR sequence was generated by site-directed mutagenesis. DNA substitution was introduced into HIF-2α 3′UTR plasmid by PCR using the following primer set: forward 5′-GTT-GCC-CTG-GCA-TTA-AGT-TAG-TTT-TAC-CCT-TGC-AG-3′ and reverse 5′-CTG-CAA-GGG-TAA-AAC-TAA-CTT-AAT-GCC-AGG-GCA-AC-3′ (the underlined nucleotide sequence indicates the substituted position). PCR reactions were conducted using a mixture of 0.7 µL Expand Long-range Enzyme Mix (Roche Diagnostics, Mannheim, Germany), 10 µL 5× Expand Long-range Buffer, 100 ng template plasmid, 100 nM primers, 3 µL DMSO, and 2.5 µL dNTPs (10 mM). PCR cycling conditions were as follows: 92 °C for 30 s, 55 °C for 1 min, 68 °C for 10 min, and a final extension at 68 °C for 10 min. PCR products were incubated with DpnI at 37 °C for 1 h to digest methylated template DNA, and then transformed into DH5α *Escherichia coli*.

For reporter assays, cells were transfected with wild-type or mutant HIF-2α 3′UTR-Luc plasmid (50 nM) and miR-con, miR-365 or anti-miR-365 (50 nM) using Lipofectamine as described in section of transfection of miRNA and siRNA. Luciferase activity was measured with a Dual-Glo Luciferase Assay system (Promega) according to the manufacturer’s instructions.

### Ribonucleoprotein immunoprecipitation assay (RNP IP assay)

RNP IP assay was performed using RIP-Assay kit for microRNA (MBL, Nagoya, Japan) according to the manufacturer’s instructions. Briefly, harvested cells were lysed by incubation with lysis buffer on ice for 10 min, followed by centrifugation at 12,000 g for 5 min at 4 °C. The supernatant was precleared by incubation with 50% protein A agarose beads slurry in lysis buffer with rotating for 1 h at 4 °C and then incubated with IgG- or Argonaute 2 (AGO2)-immobilized beads for 3 h at 4 °C. IP with IgG was used as a negative control. After centrifugation, the antibody-immobilized beads-RNP complex was washed three times with wash buffer. For isolation of miRNA and target mRNA, the Ab-RNP complex was dissolved in protein dissolving solution and RNA was precipitated by adding ice-cold 100% ethanol and incubation for 20 min at −20 °C. RNA precipitates were washed twice with ice cold 70% ethanol, dried up, and dissolved in nuclease-free water. RNA and protein from total (T; input sample taken before IP start), flow through (FT; sample from the supernatant of IP fraction), and IP fractions were prepared for qRT-PCR and western blot analysis. The levels of miR-365 and HIF-2α in RNA samples were measured as described in the section of qRT-PCR.

### Pellet culture

Primary chondrocytes and SW1353 cells were incubated with miR-365 or miR-con (50 nM) diluted in Lipofectamine for 2 h. The pellets of miR-365- or miR-con-transfected cells were formed by centrifugation 4 × 10^6^ cells at 500 g for 10 min and transferred into bottom round 96-well plate. The pellets were exposed to IL-1β (1 ng/mL) for 7 days. The medium was changed every second day for culture period and collected for measurement of MMP-13 level. For alcian blue staining, the pellets were fixed with 4% formaldehyde in PBS for 20 min at room temperature, washed three times with PBS, and stained with alcian blue solution for 2 h. Then, the pellets were rinsed with 0.1 M hydrochloric acid and washed twice with PBS.

### Statistical analysis

Data are expressed as mean ± standard deviation (SD). Statistical analysis was performed using a Mann-Whitney U test (GraphPad Prism 6). A value of *P* < 0.05 was considered statistically significant.

## Results

### Altered expression of miR-365 and HIF-2α in OA compared to normal cartilage

**Figure 1:**
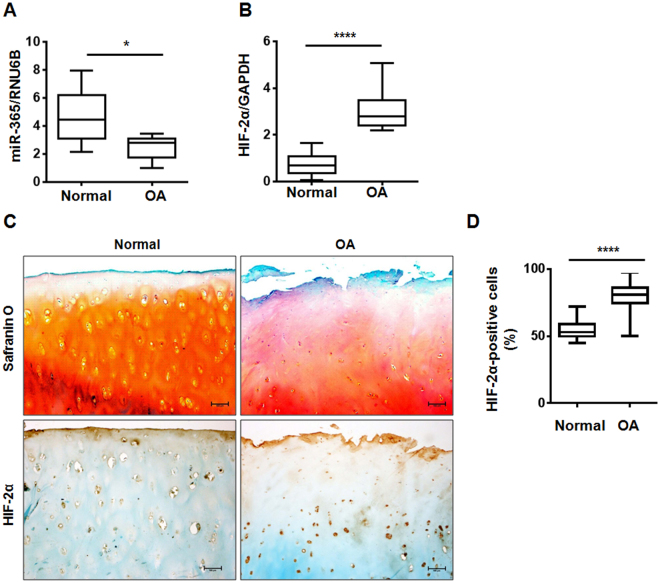
Relative expression of microRNA-365 (miR-365) and hypoxia-inducible factor-2 alpha (HIF-2α) in normal and osteoarthritic (OA) cartilage. (**A**,**B)** Relative expression of miR-365 and HIF-2α in normal and OA cartilage were assessed using (**A**) TaqMan miRNA assay and (**B**) SYBR Green-based real-time polymerase chain reaction (PCR), respectively. RNU6B and glyceraldehyde 3-phosphate dehydrogenase (GAPDH) were used as endogenous controls. *P < 0.05, ****P < 0.001 vs. normal cartilage. Data are presented as the mean ± SD of data from duplicate experiments using cartilage from different donors (n = 10). (**C**) Immunohistochemical staining for HIF-2α and safranin-O staing in normal and osteoarthritis (OA) cartilage. Expression of HIF-2α was determined by immunohistochemistry using anti-HIF-2α antibody. Sections were counterstained with methyl green. Normal cartilages were obtained from femoral head of patients with femoral neck fractures and OA cartilage from knee joints of OA patients undergoing total knee replacement surgery. Data shown are representative of results from three normal and OA cartilages. The magnification is ×100. Scale bar = 100 μm. (**D**) Quantification of HIF-2α-positive chondrocytes. Data are presented as the mean ± SD. ****P < 0.001 vs. normal cartilage.

Bioinformatics analysis of IL-1β-regulated miRNAs using miRanda, TargetScan, and PicTar revealed a putative binding site for miR-365 in the 3′UTR of the catabolic transcription factor HIF-2α. To examine the relationship between miR-365 and HIF-2α expression in OA progression, we compared the expression of miR-365 and HIF-2α in normal and OA cartilages. MiR-365 expression was significantly reduced in OA compared to normal cartilages (Fig. 1A), while HIF-2α mRNA level was increased in OA cartilage relative to controls (Fig. 1B). OA cartilages showed the loss of Safranin-O staining, fibrillation of the cartilage with fissures, and the decreased number of chondrocytes compared to normal cartilage (Fig. 1C). In addition, the level of Hif-2α-positive cells was significantly higher in OA cartilage than normal cartilage (Fig. 1C and D).

### MiR-365 and HIF-2α expression were related with IL-1β signaling pathways

**Figure 2:**
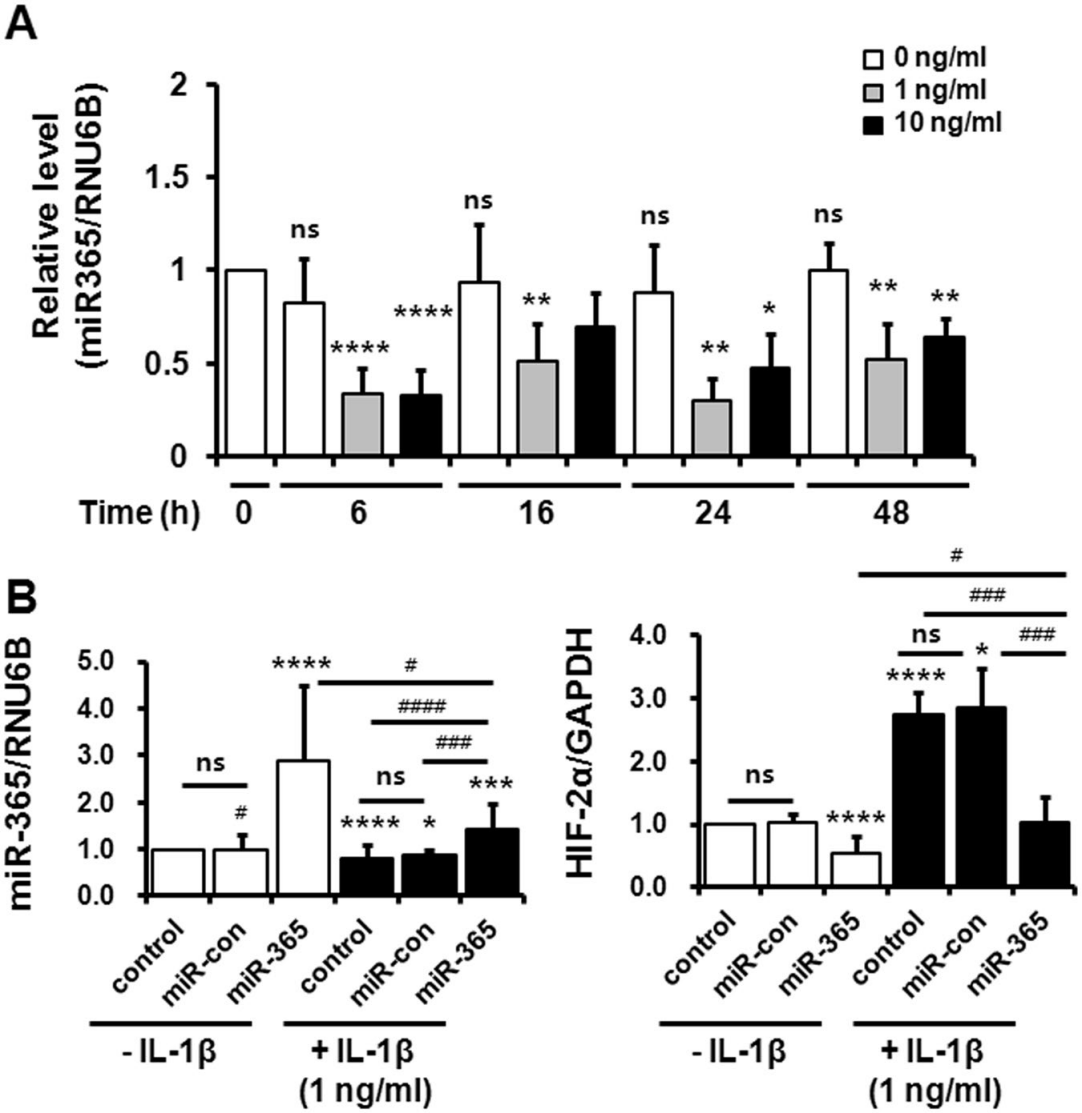
miR-365 significantly inhibits interleukin (IL)-1β-induced HIF-2α expression. (**A**) The effect of IL-1β on miR-365 expression in human OA chondrocytes. Human chondrocytes were exposed to IL-1β (1 or 10 ng/mL) for 6, 16, 24, and 48 h. Expression of miR-365 was measured using TaqMan miRNA real-time PCR assays. RNU6B was used as an endogenous control. Data are expressed as the mean ± SD of duplicate data from six independent experiments. ns, not significant, *P < 0.05, **P < 0.01 and ****P < 0.001 *vs*. IL-1β-untreated cells. (**B**) Inhibitory effect of miR-365 on IL-1β-induced HIF-2α expression. Human chondrocytes were transfected with miR-365 or a nonspecific miRNA control (miR-con) for 48 h and stimulated with IL-1β for 6 h. Expression of miR-365 and HIF-2α was measured using TaqMan miRNA and SYBR Green-based real-time PCR assays, respectively. RNU6B and GAPDH were used as endogenous controls. Data are expressed as the mean ± SD of duplicate data from more than three independent experiments. ns, not significant; *P < 0.05, ***P < 0.005, and ****P < 0.001 *vs*. IL-1β-untreated control; ^#^ *P* < 0.05, ^###^ *P* < 0.005, ^####^ *P* < 0.001 *vs*. IL-1β + miR-365-transfected cells.

To confirm that miR-365 is regulated by IL-1β^17–19^, we investigated miR-365 expression in IL-1β-treated primary chondrocytes using qRT-PCR. IL-1β treatment (1 and 10 ng/mL) significantly inhibited miR365 expression for 6, 16, 24, and 48 h compared to IL-1β-untreated cells (Fig. 2A). The suppression of miR-365 expression was not significantly different between 1 and 10 ng/mL of IL-1β except for at 16 h, when the decrease was only significant at 1 ng/mL concentration (Fig. 2A). In light of pathophysiological concentrations of IL-1β in synovial fluids from patients with OA^30,31^, we employed 1 ng/mL of IL-1β for our experiments. Having confirmed the role of IL-1β in miR-365 expression, we next examined the role of miR-365 expression on HIF-2α expression. Human primary chondrocytes were transfected with miR-365 and miR-con 48 h prior to IL-1β stimulation for 6 h. IL-1β was observed to significantly reduce miR-365 expression and increase HIF-2α expression compared to IL-1β-untreated control (Fig. 2B). However, miR-365 transfection significantly inhibited the basal expression of HIF-2α in IL-1β-untreated cells as well as IL-1β-induced HIF-2α expression (Fig. 2B). Taken together, these results demonstrate that miR-365 regulates IL-1β-induced HIF-2α expression.

**Figure 3:**
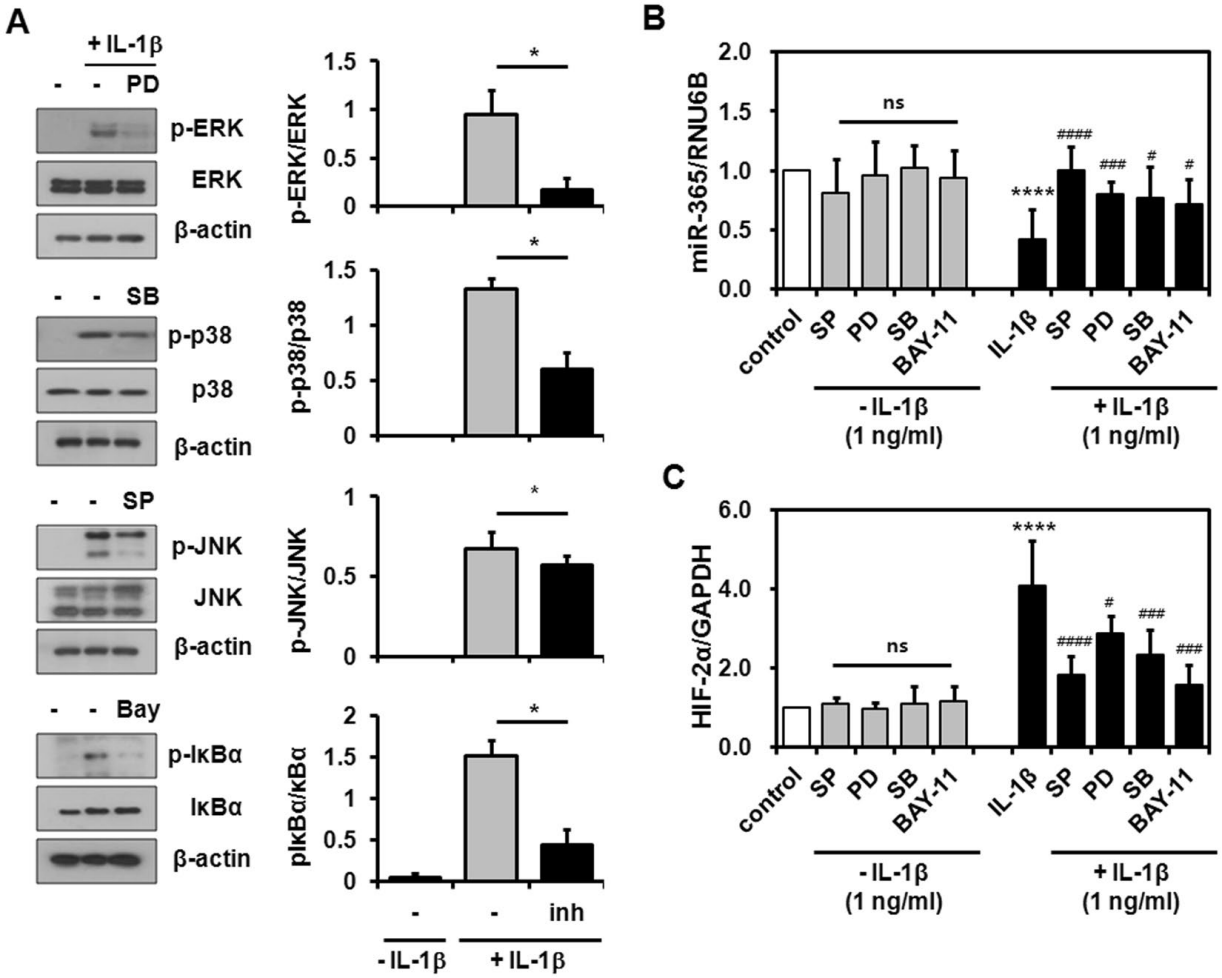
Modulation of miR-365 and HIF-2α expression by IL-1β signaling pathways. Human OA chondrocytes were incubated for 2 h with MAPK and IκBα inhibitors, including SP600125 (SP, 10 µM), PD98059 (PD, 10 µM), SB203580 (SB, 1 µM), and Bay 11-7082 (Bay, 5 µM), and stimulated with IL-1β (1 ng/mL) for 6 h. (**A**) Inactivation of MAPK and IκBα by MAPK and IκBα inhibitors at each concentration used in this experiments. (**B**,**C**) The levels of (**B**) miR-365 and (**C**) HIF-2α were measured. RNU6B and GAPDH were used as endogenous controls. Data are expressed as the mean ± SD of duplicate data from more than four independent experiments. ns, not significant, ****P < 0.001 vs. unstimulated control; ^#^ *P* < 0.05, ^###^ *P* < 0.005, ^####^ *P* < 0.001 vs. IL-1β-stimulated control.

Next, we sought to identify the IL-1β-activated signaling pathways that contribute to miR-365 and HIF-2α expression. Human chondrocytes were pretreated with SP600125 (JNK inhibitor), PD98059 (MEK-1/2 inhibitor), SB203580 (p38 MAPK inhibitor), and Bay 11-7082 (NF-κB inhibitor) for 2 h and then exposed to IL-1β for 6 h. These inhibitors at the concentrations employed in these experiments significantly inhibited the activation of MAPK and NF-κB pathways (Fig. 3A). MAPK and NF-κB inhibitors had no significant influence on the basal expression of miR-365 and HIF-2α (Fig. 3B and C). Pre-treatment with these inhibitors significantly attenuated the effects of IL-1β on miR-365 down-regulation, restoring its expression to levels at those of untreated control (Fig. 3B). At the same time, MAPK and NF-κB inhibitors significantly reduced HIF-2α expression in IL-1β-treated primary chondrocytes (Fig. 3C). Therefore, these results demonstrate that both miR-365 and HIF-2α expression are regulated by IL-1β via MAPK and NF-κB.

### HIF-2α expression was attenuated by the direct binding of miR-365 to the 3′UTR of HIF-2α mRNA

**Figure 4:**
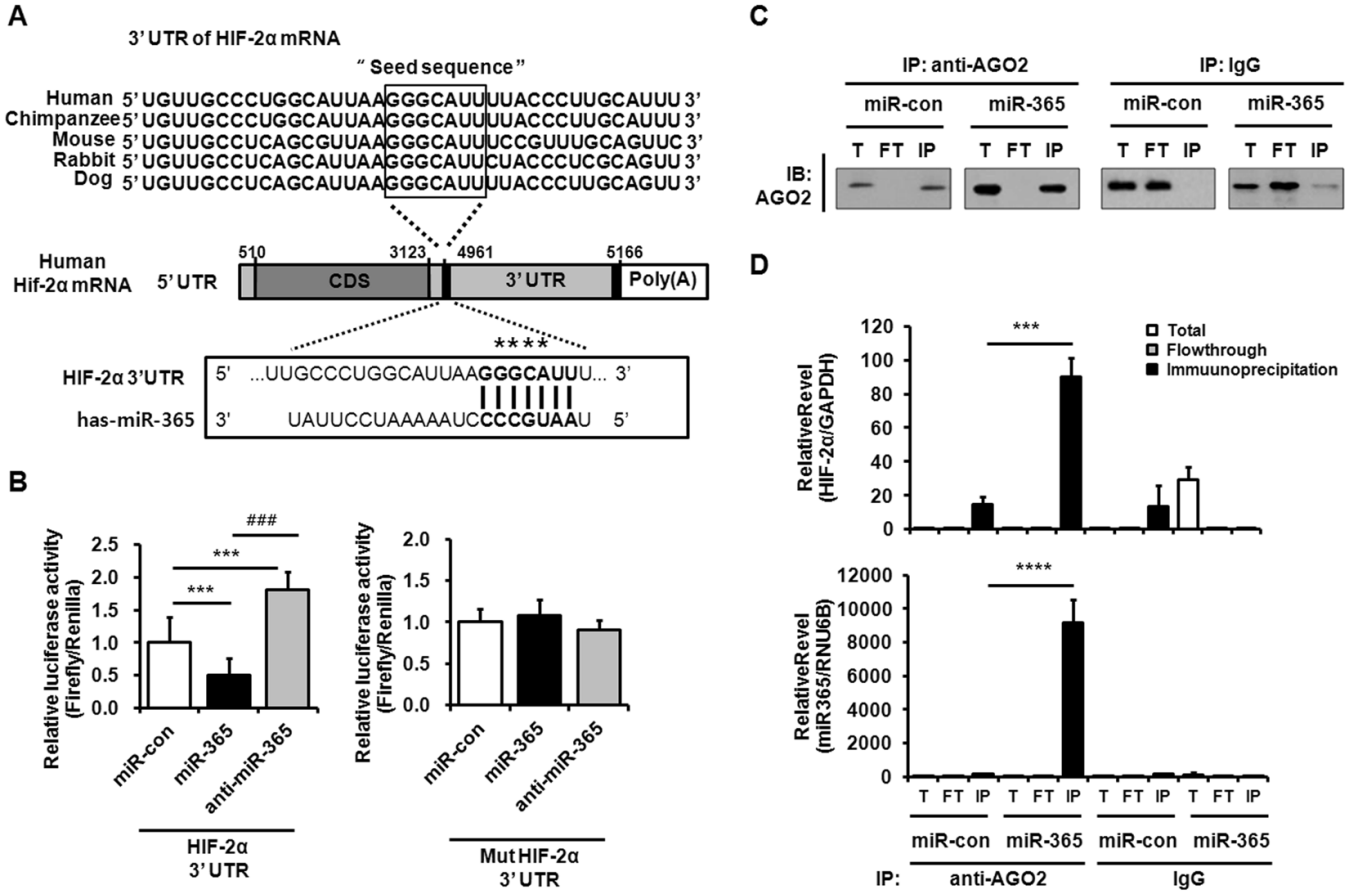
HIF-2α mRNA is a target of miR-365. (**A**) Sequence alignment of a putative miR-365 binding site within the 3′UTR of HIF-2α mRNA from human, chimpanzee, mouse, rabbit, and dog. Asterisks indicate the sequences that were changed to make mutant HIF-2α 3′UTR. CDS, coding sequence; UTR, untranslated region. (**B**) The wild-type and mutant HIF-2α 3′UTR reporter plasmid was co-transfected with miR-365, anti-miR-365 or nonspecific control microRNA (miR-con) into human chondrocytes for 48 h. Luciferase activity in each sample was determined and calculated as the ratio of reporter (firefly) to control (Renilla) activity. Data are expressed as the mean ± SD of triplicate data from more than three independent experiments. ns, not significant, ***P < 0.005 vs. miR-con-transfected cells; ^###^ *P* < 0.005 vs. miR-365-transfected cells. (**C**) AGO2-immunoprecipitation (IP) in miR-con- or miR-365-transfected chondrocytes. Primary chondrocytes were transfected with miR-con and miR-365 and stimulated with IL-1β (1 ng/mL) for 6 h. The level of miR-365 expression was quantified by qRT-PCR. The efficiency of the AGO2-IP in total (T), flowthrough (FT), and immunoprecipitation (IP) fractions of the samples was determined by western blot analysis using AGO2 antibody. IP with IgG served as a negative control. (**D**) The levels of miR-365 and HIF-2α mRNA in RNA samples from T, FT, and IP fractions of AGO2- and IgG-IP experiments were measured by qRT-PCR. ***P < 0.005 and ****P < 0.001 vs. miR-con-transfected cells.

To investigate whether miR-365 directly interacts with the 3′UTR of HIF-2α mRNA, a luciferase reporter assay was performed using a DNA construct containing the 3′UTR of HIF-2α mRNA, which harbors a putative binding site of miR-365, and is highly conserved across diverse species (Fig. 4A). miR-365 was shown to significantly inhibit HIF-2α reporter activity relative to miR-con-treated cells, whereas transfection with anti-miR-365, the antisense inhibitor of miR-365, enhanced the reporter activity of HIF-2α 3′UTR (Fig. 4B, left). Next, to confirm the association of miR-365 with HIF-2α 3′UTR, we examined the interaction of miR-365 or anti-miR-365 and a mutant reporter plasmid containing mutations in the miR-365 binding site of HIF-2α. The reporter activity data showed that a mutation in the miR-365 binding sequence removed miR-365-mediated inhibition or anti-miR-365-mediated enhancement of HIF-2α 3′UTR reporter activity (Fig. 4B, right).

To ensure that HIF-2α is the target of miR-365, we performed AGO2-RNP IP assay on miR-con- and miR-365-transfected chondrocytes. Western blot assay for checking the efficiency of RNP IP assay demonstrated that the efficiency of AGO2 immunoprecipitation (AGO2-IP) complex was comparable in miR-con- and miR-365-transfected cells (Fig. 4C). Both miR-365 and HIF-2α were abundantly presented in AGO2-IP fraction containing miR-365-RNA inducing silencing complex (RISC) complexes that were obtained using AGO2 antibody in miR-365-transfected cells compared to that of miR-con-transfected cells (Fig. 4D). Together, these data show that miR-365 suppresses HIF-2α expression by binding to the 3′UTR of HIF-2α mRNA.

### IL-1β-stimulated catabolic factors were reduced by miR-365 in chondrocytes

**Figure 5:**
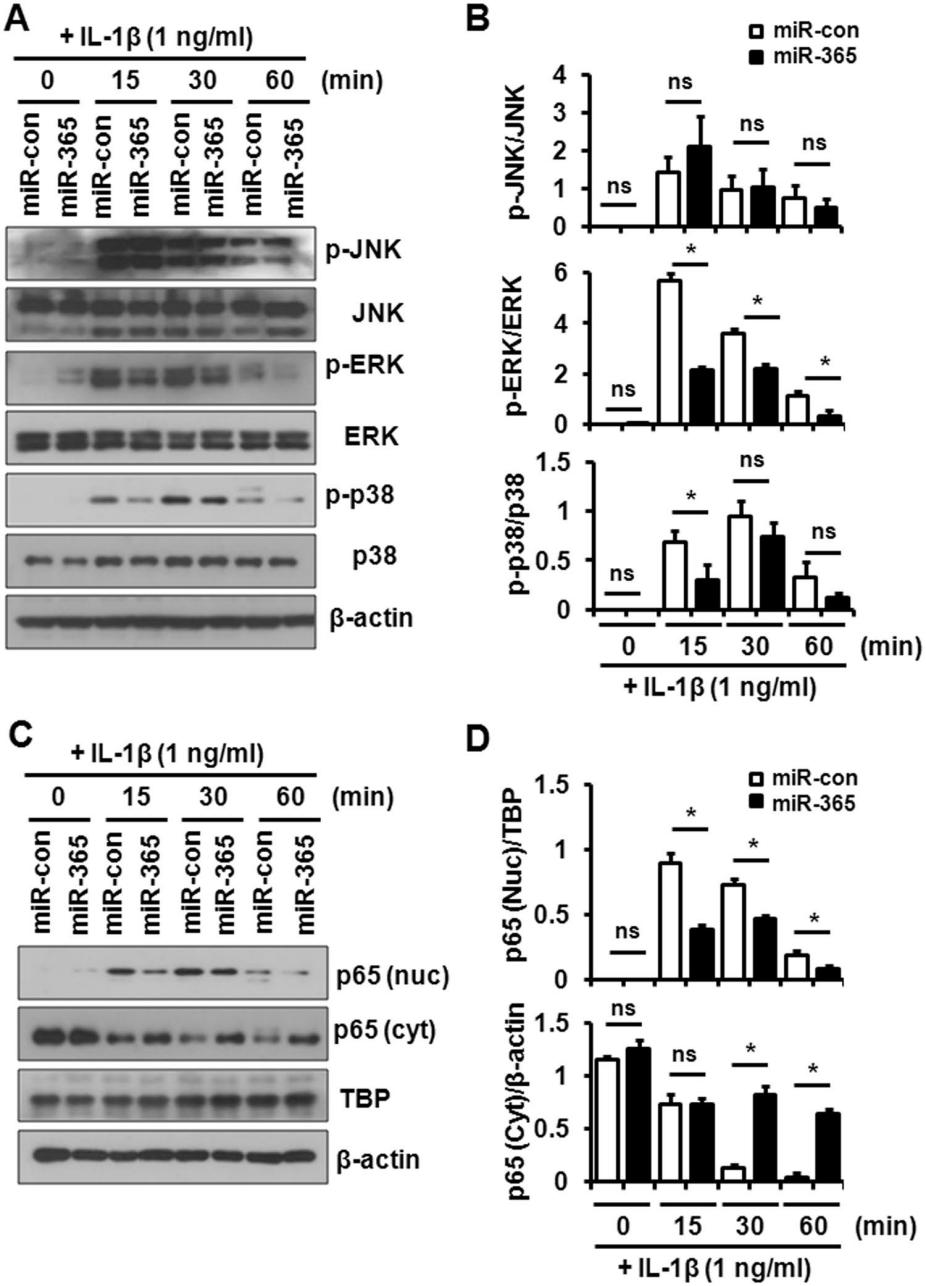
Effect of miR-365 on IL-1β-activated signaling pathways in human chondrocytes. Human OA chondrocytes were transfected with miR-365 or a nonspecific control miRNA (miR-con) for 48 h and stimulated with IL-1β (1 ng/mL) for 0, 15, 30, and 60 min. (**A**) Phosphorylation of Jun N-terminal kinase (JNK), extracellular signal-regulated kinase (ERK), and p38 and (**C**) translocation of p65 into the nucleus were analyzed by Western blot. Immunoblots shown are representative of more than three independent experiments. β-actin and TBP served as loading control. TBP, Tata-box binding protein. (**B**) The relative phosphorylation level of JNK, ERK, and p38 proteins and (**D**) the relative level of p65 in the nucleus and cytoplasm. Protein density was normalized to (**B**) the respective unphosphorylated proteins and (**D**) β-actin or TBP. Data are expressed as the mean ± SD of more than three independent experiments. ns, not significant, *P < 0.05 vs. miR-con-transfected cells.

HIF-2α is a crucial catabolic transcription factor in pro-inflammatory and OA processes^9^. Given the effects of IL-1β on miR-365 and HIF-2α expression, we examined the potential regulatory effects of miR-365 on IL-1β-activated signaling pathways. To address this question, primary chondrocytes were transfected with miR-365 and miR-con for 48 h followed by stimulation with IL-1β (1 ng/mL) for 15–60 min. Activation of MAPK and IκBα were investigated by Western blot. Transfection with miR-365 significantly inhibited IL-1β-induced phosphorylation of ERK and p38 and IL-1β-induced translocation of p65 into the nucleus in human chondrocytes, while the phosphorylation of JNK was unaltered (Fig. 5A–D).

**Figure 6:**
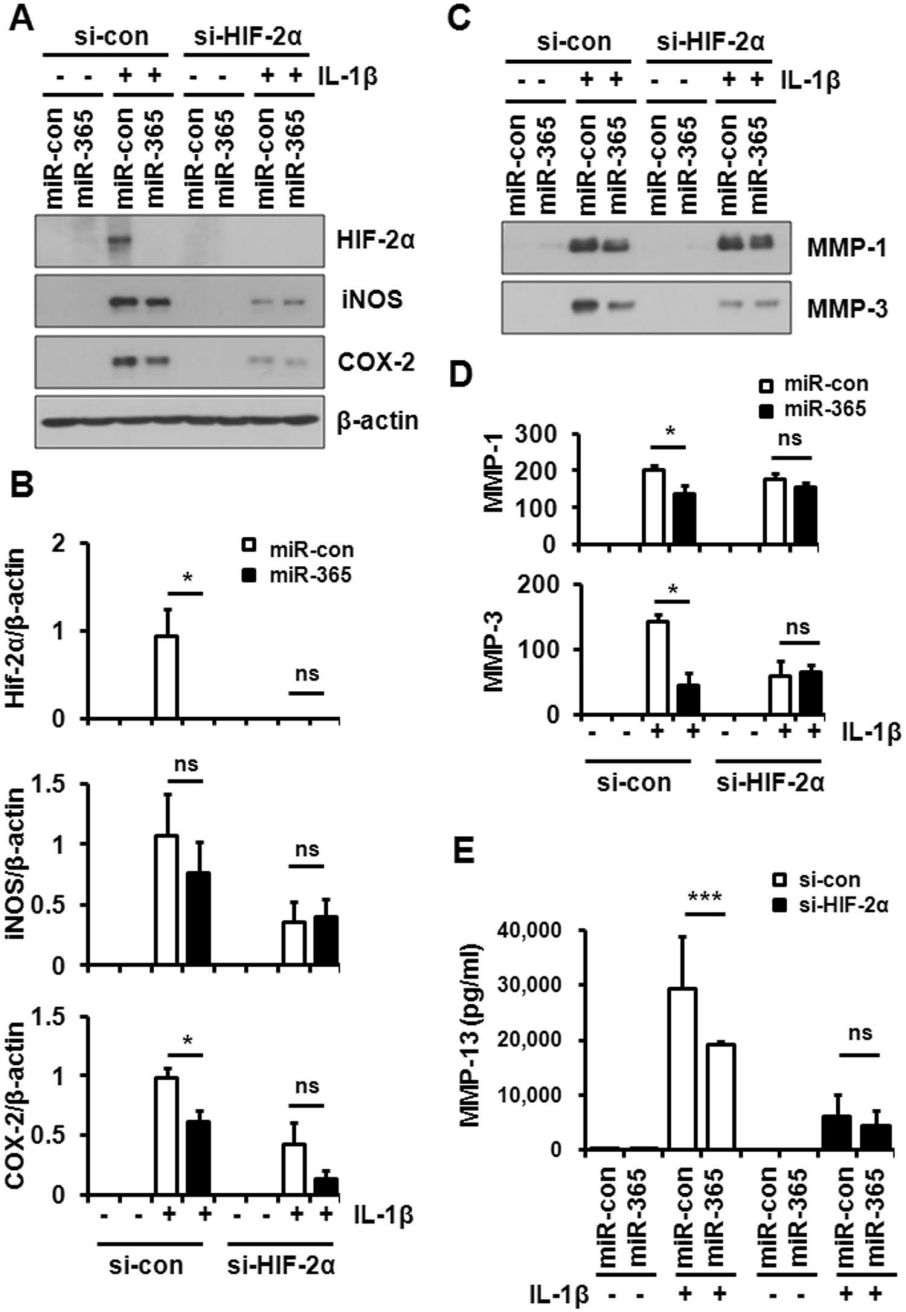
Suppression of IL-1β-mediated catabolic effect by miR-365. Human OA chondrocytes were transfected with miR-365 or miR-con and si-con or si-HIF-2α for 48 h and stimulated with IL-1β (1 ng/mL) for 24 h. (**A**) Expression of HIF-2α, inducible nitric oxide synthase (iNOS), and cyclooxygenase-2 (COX-2) in cell lysates and (**C**) matrix metalloproteinases (MMP-1 and -3) in the culture medium were analyzed by Western blot. Beta-actin (β-actin) was used as a loading control. The immunoblots shown are representative of three or more independent experiments. (**B**) The expression levels of HIF-2α, iNOS, and COX-2, and (**D**) the level of MMP-1 and -3. (**B**) Protein density was normalized to β-actin. Data are expressed as the mean ± SD of more than three independent experiments. ns, not significant, *P < 0.05 vs. miR-con-transfected cells. (**E**) MMP-13 levels in the culture medium were measured by enzyme-linked immunosorbent assay (ELISA). Data are expressed as the mean ± SD of duplicate data from more than three independent experiments. ns, not significant, *P < 0.05 vs. miR-con treated cells.

Next, we examined the expression of pro-inflammatory and catabolic proteins, including HIF-2α, COX-2, iNOS, and MMP-1, -3, and -13 by Western blot and ELISA. In line with mRNA expression data (Fig. 2B), IL-1β-induced expression of HIF-2α was significantly suppressed by miR-365 transfection (Fig. 6A). Significant reductions in COX-2, MMP-1, -3, and -13 were also observed in miR-365-transfected primary chondrocytes compared with miR-con-transfected cells (Fig. 6A–E). To confirm whether the silencing of HIF-2α counteracts miR-365-induced reduction of procatabolic effects stimulated by IL-1β, chondrocytes were transfected with si-HIF-2α and miR-365. HIF-2α was not detected in the HIF-2α-knocked down cells regardless of miR-365 transfection (Fig. 6A). In addition, IL-1β induced lower expression of iNOS, COX-2, MMP-3 and -13, except for MMP-1, in HIF-2α-knocked down chondrocytes compared to si-con-transfected cells (Fig. 6A–E). Furthermore, miR-365 transfection had no significant effect on COX-2 and iNOS as well as MMP-1, -3, and -13 expressions in the HIF-2α-knocked down chondrocytes (Fig. 6A–C). These results demonstrate that miR-365 functions as a regulator of IL-1β-induced pro-inflammatory and catabolic factors via modulation of HIF-2α.

### miR-365 prevented IL-1β-induced MMP-13 and loss of extracellular matrix (ECM) in pellet culture of chondrocytes

**Figure 7:**
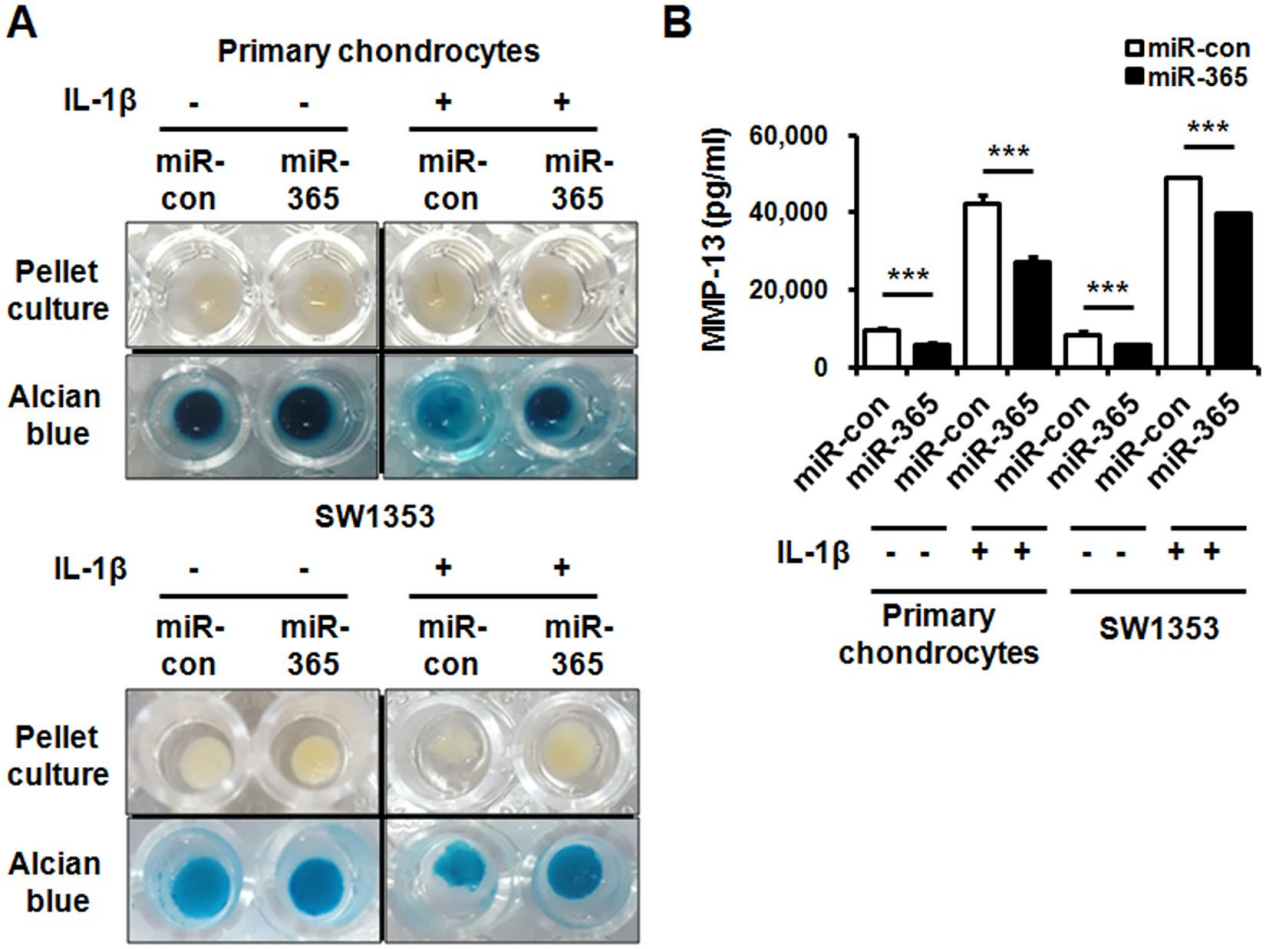
Suppression of loss of ECM and expression of MMP-13 by miR-365 in the pellet culture. (**A**) Alcian blue staining following 3D pellet culture of primary chondrocytes and SW1353 cells. Human primary chondrocytes and SW1353 cells were transfected with miR-con or miR-365 and the pellets formed by centrifugation were stimulated with IL-1β (1 ng/mL) for 7 days. The medium containing IL-1β was changed every second day for culture period. The pellets were stained with Alcian blue. (**B**) The levels of MMP-13 released into the medium from the pellet culture were measured by enzyme-linked immunosorbent assay (ELISA). Data are expressed as the mean ± SD of duplicate data from three independent experiments. ns, not significant, ***P < 0.005 vs. miR-con- or miR-con + IL-1β-treated cells.

To investigate whether miR-365 suppresses loss of ECM induced by IL-1β, the pellet culture of miR-365-transfected chondrocytes was performed in DMEM containing IL-1β for 7 days. The pellet morphology data demonstrated that no significant changes were observed in the size of miR-con- and miR-365-transfected primary chondrocyte and SW1353 cell pellets in the absence of IL-1β (Fig. 7A). However, miR-365 suppressed the reduction of pellet size induced by IL-1β, which significantly reduced the size of miR-con-transfected pellet (Fig. 7A). In addition, alcian blue staining for ECM showed that IL-1β caused fragile cell masses and weak staining in miR-con-transfected group, whereas miR-365 significantly prevented IL-1β-induced changes in cell mass and ECM matrix (Fig. 7A). In addition, the concentrations of MMP-13 protein released from pellet culture of primary chondrocytes and SW1353 cells were measured by ELISA. As expected, the secreted MMP-13 levels in both IL-1β-untreated and IL-1β-stimulated pellet culture of primary chondrocytes and SW1353 cells were significantly suppressed by miR-365 transfection compared to the miR-con transfected groups (Fig. 7B). Taken together, these data demonstrated that miR-365 significantly suppressed IL-1β-induced catabolic effects in 3D culture of primary chondrocytes.

## Discussion

Inflammatory and catabolic processes triggered by IL-1β play an important role in the destruction of cartilage in chronic joint diseases such as OA or RA. In the present study, we investigated miRNA-mediated post-transcriptional control of a key mediator of OA, HIF-2α, which is regulated by IL-1β in human chondrocytes. Using an unbiased bioinformatics analysis of IL-1β-stimulated miRNAs, we identified a putative binding site for miR-365 in the 3′UTR of the catabolic transcription factor HIF-2α. MiR-365 expression was markedly suppressed in OA compared to normal cartilage, and was shown to actively suppress the expression of various HIF-2α-regulated catabolic factors in chondrocytes.

Mammalian cells sense oxygen depletion and modulate the expression of various genes to maintain oxygen homeostasis. Chondrocytes located in the articular cartilage, an avascular tissue, are particularly susceptible to low oxygen pressure, with hypoxia regarded as an important factor for chondrocyte differentiation and cartilage matrix synthesis. Previous reports have shown that chondrocyte gene expression profiles are significantly influenced by the HIF pathway, which may be activated by two structurally similar isoforms, HIF-1α and HIF-2α, which dimerize with HIF-1β. HIF-1α induces cartilage formation and inhibits chondrocyte hypertrophy and endochondral ossification through the BMP2 signaling pathway^32,33^. Targets of this pathway include a diverse set of genes, including VEGF-A, IGFBP-3, iNOS, and leptin, all of which are enhanced during OA progression^34–37^. Outside of hypoxia, mechanical stress and inflammatory cytokines have been shown to activate HIF-1 in OA cartilage^34^. Induction of HIF-2α by IL-1β was reported in articular chondrocytes as well as cardiac myocytes^9,38^. Compared to cartilage, HIF-2α regulated by IL-1β in cardiac myocytes play a role in the adaptation of the cardiac myocytes during heart failure ameliorating cardiac function. Thus, the role IL-1 regulated HIF-2α may vary according to cells and tissue. In contrast, HIF-2α-mediated signaling in cartilage leads to tissue destruction and progression of OA. HIF-2α activation has been shown to stimulate chondrocyte hypertrophy and endochondral ossification, while suppressing autophagy^39^. In a Japanese population-based cohort study, a single nucleotide polymorphism in the promoter region of human HIF-2α was strongly associated with knee OA^12^. Furthermore, HIF-2α-heterozygous deleted mice are protected from experimental OA induced by destabilization of the medial meniscus or collagenase injection, suggesting HIF-2α is a catabolic regulator responsible for OA cartilage destruction^9,12^. Furthermore, intra-articular injection of an HIF-2α overexpression vector was sufficient to induce OA development in a mouse model of knee OA^9^. Finally, nicotinamide phosphoribosyltransferase, a catabolic regulator of OA, was found to be modulated by HIF-2α^40^. The data presented here indicated an increase in HIF-2α expression in OA cartilage, suggesting that HIF-2α is a crucial mediator of cartilage destruction and OA development.

Increasing evidence has showed that alteration of miRNA expression is linked to the pathogenesis of many diseases, including OA^15,16^. It has been reported that miR-365 plays a role in a variety of cellular processes including cell proliferation, apoptosis, and differentiation. MiR-365 was shown to confer anti-tumor activity in multiple human cancer cell lines by means of cell cycle regulation and induction of apoptosis^41–43^. It was also reported that miR-365 regulates IL-6 expression via the MAPK/ERK pathway in HEK293 and Hela cells, while enhanced expression of IL-6 in macrophages of patients with pulmonary tuberculosis is associated with downregulation of miR-365^44,45^. MiR-365 was identified as a mechanoresponsive microRNA in primary chicken chondrocytes cultured in 3-dimensional collagen scaffoldings under cyclic loading^28^. MiR-365 downregulated HDAC4, resulting in decreased chondrocyte hypertrophy indicating that miR-365 is an important regulator of both chondrocyte hypertrophy and differentiation^28^. Hydrostatic pressure suppressed the expression of miR-365 and downregulation of HDAC4 in OA chondrocytes, possibly leading to decrease in catabolic activities of chondrocytes^29,46^. In contrast to our result, previous reports showed that miR-365 was up-regulated by IL-1β stimulation and in rat anterior cruciate ligament (ACL) surgery induced OA cartilage as well as human OA cartilage^29,46^. However, our result showed that both at 1 and 10 ng/mL IL-1β decreased miR-365 expression which persisted throughout the experiment duration spanning from 1 to 48 hours. The average age of cartilage donors (approximately 71 yrs vs 62.7 or 56.2 yrs), and control specimens classification (cartilage from the femoral head of patients with femur neck fracture and no known history of OA or RA vs cartilage (normal looking non-loaded area) from patient with primary or traumatic OA) might have caused the discrepancy in miR-365 expression in OA cartilage. Our luciferase assay confirmed that miR-365 directly interacts with the 3′UTR of HIF-2α mRNA. Downregulation of miR-365 resulted in the increased expression of HIF-2α and MMP-13 as well as a variety of other catabolic genes, including COX2, iNOS, and MMP-1, and -3, all of which are under the control of HIF-2α^9^. These results suggest that miR-365 alleviates IL-1β-induced catabolism by modulating HIF-2α at the posttranscriptional level, and through cross-regulation of MAPK-NF-kB signaling. A recent study reported that the clinically approved HDAC inhibitor Vorinostat specifically increases HIF-2α in soft tissue sarcoma cell^47^. Whether the regulation of HIF-2α by HDAC4 in chondrocytes is an upstream event of the catabolic regulation by miR-365 is a subject of further research. Considering the role of HIF-2α in the regulation of cartilage degradation, these data strongly implicate miR-365 as a potential therapeutic target for the treatment of OA, though significant obstacles still remain in terms of miRNA delivery and possible off-target effects due to redundant biological targets of a single miRNA.

In summary, we demonstrate that miR-365 levels were significantly suppressed in OA cartilage, and that IL-1β decreased the level of miR-365 in articular chondrocytes through activation of MAPK and NF-κB signaling pathways. miR-365 suppressed IL-1β-mediated catabolic responses in monolayer and 3D culture of articular chondrocytes, with concurrent regulation of HIF-2α expression, suggesting that miR-365 could be a useful target for OA therapy.
